# Big data and tactical analysis in elite soccer: future challenges and opportunities for sports science

**DOI:** 10.1186/s40064-016-3108-2

**Published:** 2016-08-24

**Authors:** Robert Rein, Daniel Memmert

**Affiliations:** Institute of Cognition and Team/Racket Sport Research, German Sport University Cologne, Am Sportpark Müngersdorf 6, 50933 Cologne, Germany

**Keywords:** Big data, Sports performance, Sports analytics, Machine learning, Simulation, Spatiotemporal data, Neural networks, Deep learning, Quantified self

## Abstract

Until recently tactical analysis in elite soccer were based on observational data using variables which discard most contextual information. Analyses of team tactics require however detailed data from various sources including technical skill, individual physiological performance, and team formations among others to represent the complex processes underlying team tactical behavior. Accordingly, little is known about how these different factors influence team tactical behavior in elite soccer. In parts, this has also been due to the lack of available data. Increasingly however, detailed game logs obtained through next-generation tracking technologies in addition to physiological training data collected through novel miniature sensor technologies have become available for research. This leads however to the opposite problem where the shear amount of data becomes an obstacle in itself as methodological guidelines as well as theoretical modelling of tactical decision making in team sports is lacking. The present paper discusses how big data and modern machine learning technologies may help to address these issues and aid in developing a theoretical model for tactical decision making in team sports. As experience from medical applications show, significant organizational obstacles regarding data governance and access to technologies must be overcome first. The present work discusses these issues with respect to tactical analyses in elite soccer and propose a technological stack which aims to introduce big data technologies into elite soccer research. The proposed approach could also serve as a guideline for other sports science domains as increasing data size is becoming a wide-spread phenomenon.

Tactics are a central component for success in modern elite soccer. Yet until recently, there have been few detailed scientific investigations of team tactics. One reason in this regard has been the lack of available, relevant data. With the development of advanced tracking technologies this situation has changed recently. Instead, now the amount of available data is becoming increasingly difficult to manage. In the present article we discuss how recent developments of big data technologies from industrial data analytics domains address these problems. Further, the present work provide an overview how big data technologies may provide new opportunities to study tactical behavior in elite soccer and what future challengers lie ahead.

## Soccer tactics background

According to the Oxford dictionary, tactics describe “an action or strategy carefully planned to achieve a specific end”. Regarding competitive soccer, naturally the aim the end of the activity is to win the game. Choosing an appropriate tactic is therefore crucial for every pre-game preparation (Carling et al. 2005b; Kannekens et al. 2011; Sampaio and Macas 2012; Yiannakos and Armatas 2006). Regarding the definition of tactics Gréhaigne and Godbout (1995) introduced a distinction between the strategy and tactics. Here, the team strategy describes the decisions made before the game with respect to how the team wants to play whereas the tactic is the result of the ongoing interactions between the two opposing teams. This approach seems somewhat counter to the basic definition of the term tactics provide above. Furthermore, it is not clear how these two concepts can be clearly delineated from each other as the real-time interactions between the players will be conditioned by the a priori strategy. Following a classical practitioner’s approaches the tactic specifies how a team manages space, time, and individual actions to win a game (Fradua et al. 2013; Garganta 2009). In this context, space specifies for example were on the pitch a certain actions takes places or which area a team wants to occupy during the attack and the defense. Time in contrast describes variables like frequency of events and durations (ball possession) or how quick actions are being initiated. For example, a team could decide to have a slow buildup during attack initiate in the defense third where individual players hold the ball for longer times whereas in the attacking third only fast on-touch pass sequences are preferred. Finally, individual actions specify the type of actions which are being performed, for example turnovers, crosses and passes (Garganta 2009). This classification can be further hierarchically organized along the number of participating players into individual tactics, group tactics, team tactics, and match tactics which is also a scheme commonly referred to by soccer practitioners (Bisanz and Gerisch 1980, p.201; Carling et al. 2005a). Individual tactics describe all one-on-one events during offensive and defensive play with and without the ball. For example, the way the ball carrier is approached by a defender can be considered as part of the individual tactic. For example, the defender could immediately attack the ball carrier and put him under pressured or the defender could use a more passive approaches focusing mainly on blocking passing channels. Group tactics describe the cooperation between sub groups within a team for example the defensive block during an offside trap. Team tactics describe preferred offensive and defense team formations (e.g. 4-4-2) and the positioning of the formation on the pitch (Grunz et al. 2012). Finally, game tactics describe the team’s playing philosophy such as counter-attack or ball possession play. A recent study investigated for example ball possession regain in the German Bundesliga where the results showed that more successful teams were faster to regain ball possession after losing possession (Shafizadehkenari et al. 2014; Vogelbein et al. 2014). In summary, soccer tactics describe the microscopic and macroscopic organizational principles of the players on the pitch spanning from individual to group decision making processes.

**Fig. 1:**
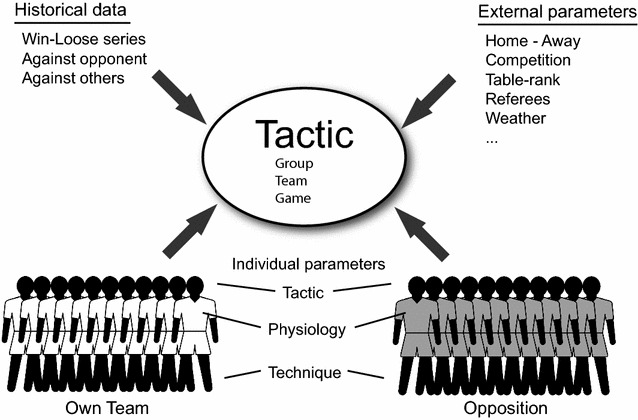
Overview of factors influencing tactics in soccer

To ensure successful execution at all tactical levels, a coach has to take into account the status of the team, the status of the opposition, as well as external factors like playing at home or even the weather (Gréhaigne and Godbout 1995; Lago 2009; Mackenzie and Cushion 2013; Sarmento et al. 2014) (compare Fig. 1). Therefore, in the following tactics refers to both the a priori decisions as well as the real-time adaptations during a game. As the two competing teams try to out-smart each other, the tactics are not constant but should be adapted according to the interactions between and within the two teams (Balagué and Torrents 2005; Garganta 2009; Grehaigne et al. 1997; Gréhaigne and Godbout 2014). For example, a player substitution by the opposition team may introduce a change in playing tactics which the coach may have to respond to be changing his teams’ tactics. Team tactics are therefore governed by a complex process resulting from a network of inter-dependent parameters (Kempe et al. 2014). Although the scheme presented above follow a hierarchical pattern the flow information in reality does go in both directions. Tactics at a higher level condition the tactics at the lower level and vice versa success of individual actions equally conditions success at a higher level (Araújo et al. 2006; Sampaio and Macas 2012). Thus, tactics can be interpreted as complex structure of composed of a new of interwoven dependencies. Accordingly, tactical analysis should reflect this complexity.

Over the years tactical decisions, like preferred playing formations or game tactics, have increased in complexity and coaches’ tactical abilities are under constant public scrutiny. Until very recently this stood somewhat in contrast to the amount of scientific investigations studying tactical decisions in elite soccer (Carling et al. 2005c; Garganta 2009; Sampaio and Macas 2012; Sarmento et al. 2014). The reason for this somewhat surprising fact may have been the lack of accessible and/or reliable data required for tactical analysis (Rampinini et al. 2007). The present gold standard to assess tactical behavior and team performance in general in elite soccer is commonly based on individual game observations (Dutt-Mazumder et al. 2011; Mackenzie and Cushion 2013). A domain expert (coach, scout) observes a game and rates the team tactics according to his personal experiences. Although usually a specific coding manual is used a general consensus regarding relevant variables is currently missing (James 2006; Sarmento et al. 2014) and data often lack objectivity and reliability (James et al. 2002). Furthermore, as game interactions are highly dynamic and contextual circumstances change continually it is under debate to what extent reliable measures are attainable in general (Lames and McGarry 2007). In addition, detailed game analyses based on observational approaches are highly time-consuming which limited their application in the past (Carling et al. 2008; James 2006). Consequently, demand for more quantitative oriented (automatic) approaches to analyze tactical behavior in elite soccer is increasing (Beetz et al. 2005; Carling et al. 2014; Lucey et al. 2013a, b; Wang et al. 2015). Thus, whereas the processes underlying tactics in elite soccer have increased over the years the scientific approaches have not quite evolved with the same speed.

In this regard, fine-grained global reporting of game event statistics for commercial audiences has seen a tremendous rise in recent years and detailed game data are routinely reported (Baca 2008; Baca et al. 2004; Sarmento et al. 2014). The reason for this increased availability of game data is largely due to progress made in player tracking technologies (Baca 2008; Carling et al. 2008; Castellano et al. 2014; D’Orazio and Leo 2010; Lu et al. 2013). Recently FIFA the governing body for international competitive soccer decided to allow the usage of wireless sensors technologies to track player positions and physiological parameters during competitions (di Salvo and Modonutti 2009). This will further increase the availability of detailed performance data from elite soccer. Thereby this has been a results of today’s common practices among professional teams to already collect physiological and tracking data during training and friendly matches to manage the training process (Bush et al. 2015; Carling et al. 2008; Ehrmann et al. 2016; Goncalves et al. 2014; Ingebrigtsen et al. 2015). At present, several different tracking systems are available in the market including vision based systems, Global Positioning Systems (GPS), and radio wave based tracking systems (Leser et al. 2011). Although data quality and reliability used to be a problem, in recent years the systems have matured to such an extent that the data is now of sufficient quality to satisfy scientific standards. Several recent overviews addressing the advantages and disadvantages between the different available systems are available in the literature (Barris and Button 2008; Buchheit et al. 2014; Carling et al. 2008; Castellano et al. 2014; D’Orazio and Leo 2010; Harley et al. 2011; Valter et al. 2006). Thus modern tracking data allows the analysis of technical, tactical and physical demands in elite soccer.

In general, a trend seems to emerge where analyses of soccer games in public media outlets are also becoming increasingly data aware. One example in this regard is the increasing number of free internet blogs reporting detailed game analyses. Using observational techniques from TV game broadcasts data as well as publicly available internet soccer databases these blogs provide novel approaches to data driven performance analysis in soccer much in the same spirits as the sabermetrics community has for American baseball during the late 90’s (Lewis 2004). Recently, investigations have emerged which used sentiment analysis from twitter feeds to identify for example high impact events during games (Buntain 2014; Yu and Wang 2015) and to predict game outcomes (Godin et al. 2014). In this regard, quantified-self initiatives may also provide future opportunities to generate valuable data for scientific investigations (Appelboom et al. 2014; Shull et al. 2014). In summary, lack of reliable data to perform tactical analysis in elite soccer is becoming less of a problem and novel data sources are continually being discovered and developed.

## Analysis of soccer tactics

Traditionally, one area which has produced a wealth of studies investigating soccer performance is with respect to the physiological demands in competitive soccer (Carling et al. 2008; Mohr et al. 2005). However, until recently few connections between physiological demands and tactical behavior in elite soccer have been made (Bloomfield et al. 2007; Drust et al. 2007; Moura et al. 2012). As was made clear in the introduction, the success for a tactics depends on the abilities of the individual players to actually implement the required actions. Obviously this requires that the players fulfill the necessary physiological requirements, for example, when playing a ball possession type of play (da Mota et al. 2016). Rampinini et al. (2007) investigated the total running distances and the time spent different running speed categories (standing to sprinting). The results showed a significant influence of the level of the opponents and the playing position (compare also Goncalves et al. 2014). Bush et al. (2015) investigated the changes in physiological performance variables in the English Premier League across several seasons and results indicated significant increases in passing event rates associated with changes in team tactics (Bush et al. 2015). Carling (2011) investigated the influence of opposition formations on physiological and skill-related performance variables and found for example increased running distances when playing against a 4-2-3-1 formation compared to a 4-4-2 formation (Carling 2011). Sampaio et al. (2014) investigated the influence of time unbalance and game pace on physiological demands during a 5-a-side small sided game were one player was dropped in either side to create an inferiority or an superiority condition. The results suggested an effect of team unbalance on the time spent in different hear rate zones suggesting that the inferior team had to work harder (Sampaio et al. 2014). In summary, these results indicate that tactical behavior and physiological variables are linked but more in-depth analyses are missing. Accordingly, at present it is unclear how to combine information about player’s physiology from training and competition with team tactics (Castellano et al. 2014) and no connections between individual technical performance and team tactics have been made so far (Hughes and Bartlett 2002).

Traditionally, tactics analyses relied on notational analysis approaches based on average statistics and tallies (Hughes and Bartlett 2002). Indicators include for example passing variables (Hughes and Franks 2005; Liu et al. 2015), ball possession (Collet 2013; Lago 2009), ball recovery (Vogelbein et al. 2014), or playing style (Tenga et al. 2010a, b). The main limitation of the traditional notational approach is that almost all contextual information is discarded, these measures have shown weak explanatory power with limited adoption by practitioners (Glazier 2015; Hughes and Bartlett 2002; Mackenzie and Cushion 2013; Nevill et al. 2008; Sarmento et al. 2014; Tenga et al. 2010a, b). To circumvent this problem increasingly multi-variate approaches are being used to retain contextual information (Fernandez-Navarro et al. 2016; Kempe et al. 2014). Almeida et al. (2016) investigated the effect of different scoring modes on ball-recovery type and location, playing configuration and defensive state in youth players. The results showed that more ball recoveries were made when a central goal was used and that most recoveries were a result of set-play in the defensive third of the pitch. Younger players also produced more elongated shapes in the playing direction whereas the older teams produced more flattened shapes with larger spread in the direction orthogonal to the playing direction (Almeida et al. 2016). Tenga et al. (2010a, b) investigated the effects of a ten different variables on score-box possession based on video data from 163 matches from the Norwegian men’s professional league in 2004. The results showed that the odds ratio for producing a score-box possession increased when the attacking team had a long possession, started their attack from the final third, or used penetrative passes against a balanced defense. However, counterattack, possession starting in the final third, long possession, long pass, and penetrative passes showed increased odds ratios against an imbalanced defense. Recently, Fernandez-Navarro et al. (2016) used 19 performance indicators to identify different playing styles. The results showed that several factors like possession directness which correlated with ball possession, sideway passes, and passes from the defensive third into the attacking third were important to identify playing styles (Fernandez-Navarro et al. 2016).

One approach which is increasingly being used to study team tactics is the team centroid method (Folgado et al. 2014; Frencken et al. 2011, 2012; Yue et al. 2008). Here the behavior of the team centroid, the geometric center of the positions of all players from a team, is used to analyze the behavior of the whole team. Results from this line of research indicate a strong coupling between team centroids during game play (Frencken et al. 2011), changes of inter-centroid distances due to pitch size variations (Duarte et al. 2012a, b; Frencken et al. 2013), and key game events like goal shots are accompanied by increased inter-team coupling variability (Frencken et al. 2012). More recently, investigation of centroid behavior has been further extended by calculating the Approximate Entropy (ApEn) (Pincus and Goldberger 1994), a non-linear time-series measurement techniques, to quantify the regularity in time-series data (Aguiar et al. 2015; Goncalves et al. 2014; Sampaio and Macas 2012). Results using ApEn analysis suggest increased centroid behavior regularity after tactical training in novice players (Duarte et al. 2012a, b; Sampaio and Macas 2012). Goncalves et al. (2014) investigated the coordination during on 11-a-side game between and within the defenders, mid-fielders, and attacker subgroups using ApEn. The results showed that players movements were more regular with respect to the centroid of their respective groups compared to the other groups. Sampaio et al. (2014) further showed that during an inferiority situation during a 5-a-side small sided game the regularity of the distance to the team centroid increased. Goncalves et al. (2016) investigated the influence of numerical imbalances between attacking and defending team in small sided games in professional and amateur players. Player numbers varied between 4 versus 3, 4 versus 5, and 4 versus 7. The results showed that in experts an increase in the number of opponents increased the regularity in team behavior with respect to the opponents. Although the application of ApEn is becoming more prominent, it still remains to be shown what this measure really represents as the regularity behavior of team centroids in itself represent a highly abstract description of team behavior. Nevertheless, team centroid measures increasingly are being used to capture team behavior and many interesting applications have been reported in the literature in recent years.

Another more recent group of approach to study team tactics focuses on the control of space. On such approach uses for example the team surface area as calculated from the convex hull which encloses all players from one team (Frencken et al. 2011; Moura et al. 2012, 2013). Results from this line of research indicates that greater surface areas are covered by the attacking compared to the defensive teams (Frencken et al. 2011; Moura et al. 2012). Similar, more experienced players also cover a greater area compared to less experienced players (Duarte et al. 2012a, b; Olthof et al. 2015). Fradua et al. (2013) investigated the individual player area during 11-a-side matches by calculating the largest rectangle enclosing all field players divided by the number of players. The results showed that individual playing areas become smaller when the ball moved into the central pitch area. Another approach uses Voronoi-diagrams to investigate space control (Nakanishi et al. 2008). Here the controlled space is determined using the location and distances between individual players to determine the controlled space. Results using Voronoi-diagrams show similar results compared to the team surface area approach (Fonseca et al. 2012; Fujimura and Sugihara 2005; Gudmundsson and Wolle 2014; Kim 2004; Taki and Hasegawa 2000) Finally, another approach is based on the determination of numerical superiority in a particular pitch area (Silva et al. 2014). Together these results indicate that space control is a central aspect of soccer tactics and further highlight the interactive nature underlying soccer games (Duarte et al. 2013; Garganta 2009; Grehaigne et al. 1997; Tenga et al. 2010a, b).

Another emerging analysis approach to study team tactics studies investigates team passing behavior using network approaches (Watts and Strogatz 1998). The basic rationale of this approach is to model the players of a team as nodes and the passes occurring between them as weighted vertices where the number of passes between two players determine the weights (Duarte et al. 2012a, b; Passos et al. 2011). This representation of team passing behavior allows to easily identify key players within in a team as they display more connection to other vertices accompanied by greater vertex weights (Gama et al. 2014; Passos et al. 2011). Recent network analyses which included next to the player information also pass position information were able to predict game outcomes and the final ranking of the top teams using a K-Nearest Neighbor classifier (Cintia et al. 2015). Similar, Wang et al. (2015) used Bayesian latent model approach applied to passing network and passing position information from 241 games from the Spanish First (2013–2014). The obtained model was able to automatically identify different tactical patterns across teams. By combining the obtained tactical information with attacking success the authors were further able to show which specific tactical patterns were more efficient across teams. By investigating the contributions by the individual players to each tactical pattern the authors were further able to determine individual contributions by the players to each tactical pattern (Wang et al. 2015). Together these results suggest that players interactions mediated through passing behavior in combination with spatial information provides an interesting new approaches to analyze tactical behavior in elite soccer thereby providing much more information compared to traditional notational analysis approaches.

Increasingly tactical decision making in elite soccer is also investigated using machine learning (ML) algorithms based on game position data (Bialkowski et al. 2014a, b; Fernando et al. 2015; Xinyu et al. 2013). Machine learning algorithms allow to identify specific data patterns in large datasets by building an a priori unknown model from the data (Haykin 2009; Jordan and Mitchell 2015; Waljee and Higgins 2010). Although this approach has been discussed in sports research for some time (Bartlett 2004; Borrie et al. 2002; Nevill et al. 2008) only recently successful applications become more prevalent (Bartlett 2004; Lucey et al. 2013a, b). For example, application of an expectation maximization algorithm with position data from an entire English Premier League season allowed the automatic identification of team formations (Bialkowski et al. 2014a, b; Lucey et al. 2013a, b). The results further showed that teams used more defensive formations during away games (Bialkowski et al. 2014a, b). The authors used a two-step algorithm where the formations were identified only after each player was assigned a specific role. This approach allowed the authors to circumvent the problem that the player’ roles are not constant throughout the game but change according to the context which precludes the possibility to simply use the id of each individual player to identify team formations (Bialkowski et al. 2014a; Lucey et al. 2013a, b). Knauf et al. (2016) used a spatio-temporal kernel algorithm to cluster trajectories which allowed automatic differentiated game initiation and scoring opportunities from position data. Pairwise similarities between trajectories during attacking phases were compared using a specific metric and subsequently a clustering algorithms grouped the trajectories into clusters. Again, one of the underlying features of the algorithm used by the authors is that the comparison between trajectories is invariant to permutations between players (Knauf et al. 2016). Using spatial tracking data, Kihwan et al. (2010) applied a temporal kernel method to predict the location of the ball on the pitch. By calculating a flow-field from the running directions of the players the authors were able to determine convergence points of flow-field which predicted future positions of the ball with good agreement (Kihwan et al. 2010). Hirano and Tsumoto (2005) used a multiscale comparison technique with combined event data type and event location data to automatically identify reoccurring attacking sequences leading to a goal. The multiscale comparison technique allowed to compare event sequences of varying length with each other. For example, in the spatial-kernel method this problem has been resolved by time-normalizing the data (Knauf et al. 2016). Similar, Fernando et al. (2015) were able to differentiate attacking plays across teams using cluster analysis of game sequences (compare also Xinyu et al. 2013). Recently, Montoliu et al. (2015) applied a Bag-of-Words algorithm to coding soccer game video snippets followed by a Random Forest classifier to identify game play patterns. The authors divided the pitch into ten areas and calculated the optical flow representing the moving direction of players during short video sequences extracted from two complete soccer game recording. Thus, the application relied on the pre-segmentation of the raw video data by experts (Montoliu et al. 2015).

A second group of ML approaches featuring prominent in the soccer literature uses neural network modeling (compare Dutt-Mazumder et al. 2011 for a comprehensive overview). Here, in particular Kohonen Feature Maps (KFM) have been used to study tactical patterns (Barton et al. 2006; Bauer and Schöllhorn 1997; Dutt-Mazumder et al. 2011; Kohonen 1990, 2001; Lees and Barton 2003). For example, Grunz et al. (2012) used a Hierarchically Dynamically Controlled Network KFM (Perl 2002, 2004; Perl and Weber 2004) to automatically identify team formations (Grunz et al. 2012; Kempe et al. 2015; Memmert and Perl 2009). In summary, numerous machine learning studies of have used soccer data to study tactical decision making with little guidance for non-experts. Common to these approaches is that mostly a certain facet of team tactics, predominantly team formations, was investigated. Accordingly, information how to combine the information across tactical domains (Fig. 1) is lacking currently (Garganta 2009; Glazier 2015). For example it is not clear how group formations interact with the individual technical and tactical skills of players. As it is clear that different tactical positions within a team have different physiological demands there has been no research addressing how this information can be used in combination with tactical formations used by the attacking and defensive teams (Carling et al. 2008). Furthermore, with respect to the tactics hierarchy introduced in the introduction (compare also Fig. 1) the presented approaches work at the team tactics level. Accordingly, how team formations influence group tactics of subgroups and individual tactics has not been investigated so far. An interesting side-note of the presented studies is the fact that most ML soccer analyses are performed by computer scientist research group with little apparent involvement by sports scientists.

This short overview shows that although many interesting analyses are available what is lacking is a conceptual connection between them. Accordingly, it appears that the main obstacle to study team tactics stems from the lack of a theoretical model (Garganta 2009; Glazier 2015; Mackenzie and Cushion 2013). One model which has been repeatedly proposed in the literature is based on a Dynamic system theoretical framework (Duarte et al. 2012a, b; Duarte et al. 2013; Garganta 2009; McGarry et al. 2002; Reed and Hughes 2006; Ric et al. 2016). However, although this approach merits great potential, at present already the basic definition of a relevant phase space is lacking. In the dynamic systems theoretical approaches, the phase space constitutes a key concept which describes a theoretical abstractions describing mathematically a space where the system resides in and which enable to capture the dynamics of the system in a meaningful manner (Nevill et al. 2008; Vogel 1999). Current suggestions regarding appropriate phase space variables in team game vary widely (Duarte et al. 2012a, b; Gréhaigne 2011; Grehaigne et al. 1997; Gréhaigne and Godbout 2014; Lames and McGarry 2007). In this regard, a common approach for example is to use the relative phase as a measure to capture coordination phenomena between players (Duarte et al. 2013; Goncalves et al. 2014; Sampaio and Macas 2012). Relative phase approaches stem from the domain of physical dynamical systems were oscillators typically constitute the building blocks of the systems (Pikovsky et al. 2003). Accordingly, the question of whether an oscillator assumption is justified to model team games is an open question at present. Modeling efforts of soccer games as a dynamic system which go beyond a purely phenomenological description are therefore not available at present.

The lack of a higher-order description about soccer team dynamics also prevents the current analytical approaches from making a real impact with practitioners (Carling et al. 2008; Lames and McGarry 2007; Nevill et al. 2008). One of the challenges for tactical match analysis in elite soccer will be to work towards an explanatory theoretical model which is able to integrate information from various domains including tactics, physiology, and motor skills (Garganta 2009; Sarmento et al. 2014) (compare Fig. 1). In this regard, new approaches in Artificial Intelligence (AI) research (Bishop 2013; Gibney 2016; Jones 2014; LeCun et al. 2015) may provide promising avenues towards the development of a theoretical model of tactical decision making in elite soccer. In particular, so-called deep learning networks are becoming increasingly powerful in modeling domains previously considered computational intractable (Hinton and Salakhutdinov 2006; LeCun et al. 2015; Xue-wen and Xiaotong 2014). However, these approaches rely on large training datasets to determine network parameters (Jones 2014; Xue-wen and Xiaotong 2014), which at present have not been used in tactical analyses in soccer. In this regard, recent machine learning models using neural networks have been extended such to allow to incorporate a priori information into the models (Bishop 2013). This might be of great relevance to develop novel approach to model team tactical behaviors as for example insights gained from the studies summarized above might be used to constrain network modeling efforts and at the same time allowing the connection between physiological, tactical and skill related information. Accordingly, modern algorithm from AI might prove highly useful for tactical analysis in elite soccer and fulfill previous proposals (Dutt-Mazumder et al. 2011).

## Big data and soccer tactics

A potential solution with respect to model building and the combination various data sources might present itself through the recent rise of big data technologies which has been already suggested as shaping the future of performance analysis in elite soccer (Cassimally 2012; Kasabian 2014; Lohr 2012; Medeiros 2014; Norton 2014). As the phenomenon of big data is relatively recent first a definition of the relevant concepts will be provided. Surprisingly, no universally agreed definition of big data is available and big data is rather described by its characteristics (Baro et al. 2015; Noor et al. 2015; Romanillos et al. 2016). Accordingly, big data is characterized using the so-called three V’s: (1) Volume, (2) Variety and (3) Velocity (Noor et al. 2015; Xue-wen and Xiaotong 2014). Volume describes the magnitude of the data, Variety refers to the heterogeneity of data, and Velocity characterizes the data production rate (Noor et al. 2015). With respect to tactical analytics in soccer these concept can be mapped in the following way: (1) Volume refers to the size of datasets in soccer. For example, a current dataset for positional data typically encoded using Extensible Markup Language (XML) ranges between 86 and 300 megabytes (mb). Thus, storing position, event and video data from a single complete Bundesliga season results in 400 gigabytes of tracking data. Accordingly the data volume increases with the addition of other sources including for example physiological or event data. By itself this is far from the petabyte data sizes commonly associated with big data (Pääkkönen and Pakkala 2015), yet the main problem is to provide structured access to the data. Common solutions using Excel sheets do not scale well with these data. Big data technologies in contrast provide specific solutions for storing such data sets and make them accessible through specific user interfaces and application programming interfaces (API). (2) Variety refers to different data formats and data sources. Variety can be further distinguished into: (a) structured, (b) semi-structured, and (c) unstructured data. Structured data has a clearly predefined schema describing the data. Structured data allows simple navigation and searching through the data where a relational database system is the canonical example. In contrast, unstructured data lacks a definite schema with video data and text messages being typical examples. Accordingly, semi-structured data falls in between these two extremes and consists of data which lacks a pre-defined structure but may has a variable schema which is often part of the data itself (Sint et al. 2009). Current XML data types used for tracking data are examples in this regard (IPTC 2001). Thus, in soccer data variety refers to position, video, fitness, training, skill performance, and notational meta-data next to health records and crowd data from blogs. As data access and data processing patterns vary across data types, big data technologies provide specific solutions to combine the information distributed across such datasets. (3) Velocity describes the speed with which novel data is being generated. In soccer, the velocity varies widely between real-time streams from physiological and positional data to delayed data from notational analysis during training and competition. Big data technologies specifically address how to process and store high velocity data. In summary, all three key concepts characterizing big data are highly relevant with respect to tactical analysis in elite soccer and big data technological stacks provide specific solutions to address each of these areas.

**Fig. 2:**
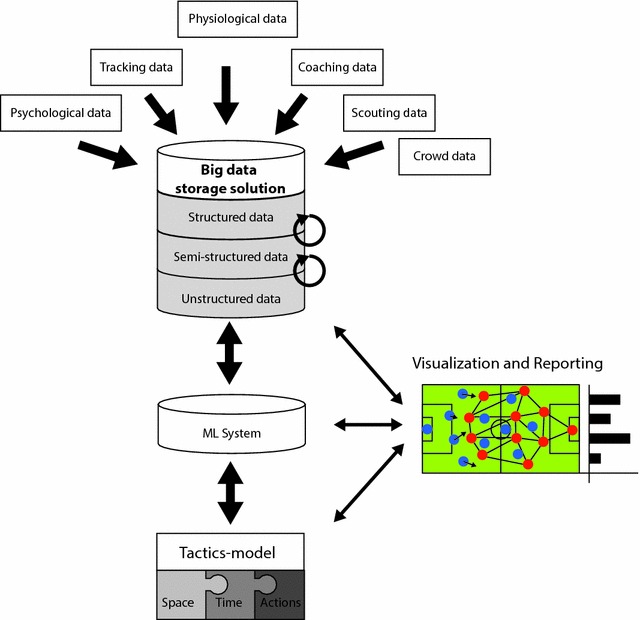
Big data technological stack for tactical analysis in elite soccer

A candidate big data soccer technological stack for soccer tactics analyses should be organized along several levels (compare Fig. 2). First, the necessary infrastructure to collect the data is required spanning physiological and tracking data in addition to video and observational data. Second, a storage system is required allowing efficient data storage and access. Finally, a processing pipeline has to be established to extract relevant information from the data and to subsequently merge the information to build an explanatory and/or predictive model (Coutts 2014). For all these processing levels reporting and visualization capabilities are needed to monitor the different processing steps and communicate the results. Unfortunately, there is no one-to-one mapping between these different components and available technologies. However an in-depth discussion of specific technological solutions is beyond the scope of the present article and more specialized literature is referred to (Noor et al. 2015; Pääkkönen and Pakkala 2015; Sitto and Presser 2015).

Yet, what immediately becomes clear from Fig. 2 is that a significant amount of expertise is needed in order to establish such a system. One area which is facing similar challenges in this respect is the medical health sector (Noor et al. 2015; Toga et al. 2015; Zhang et al. 2015). In the medical area a so-called personalized (stratified) medicine is increasingly seen is a key are of research to improve current practices (Hood et al. 2015; Kostkova et al. 2016; Zhang et al. 2015). Thereby, for personalized medicine to become realizable big data technologies are needed. One key problem in this area is how data is stored and shared across institutions. At present health data is collected and held by government, commercial and public research institutions. This leads to sever limitations with respect to access and data sharing possibilities across these entities due to privacy and security issues (Costa 2014; Kong and Xiao 2015; Kostkova et al. 2016; Toga and Dinov 2015). This also applies to soccer data where data is collected by commercial institutions, private clubs, and public research institutions. Accordingly, privacy issues have to be addressed as for example detailed profiles about individual players might have significant career implications and professional soccer teams may be reluctant to share data and possibly forfeit competitive advantages. Thus, data governance issues must be resolved before big data approaches may become viable for soccer research potentially. In the medical sector varies solutions are being investigated including standardized open privacy protection mechanisms which encrypts individual data items (Kong and Xiao 2015). Nevertheless, even when access is made available, researchers face the problem that data processing is highly complex and not manageable using common processing pipelines. Experiences from the biomedical sectors shows that in particular smaller research groups lack the required expertise and funding to build the required processing and analysis infrastructures (Bishop 2013; Goecks et al. 2010; Lynch 2008; Marx 2013; Noor et al. 2015; Sitto and Presser 2015). At present, it is also not clear how to ensure that technologies and procedures are made available to researchers lacking the required computer science expertise to build data pipelines of their own. This is already a problem with respect to many of the ML techniques described above.

As computational approaches increasingly become more complex reproducibility issue will also become more important as the development of novel algorithmic approach will become the focus of future publication results (Mesirov 2010). In this regard, efforts from biomedical research like the Galaxy project (Goecks et al. 2010) may provide a model solution for future big data technologies in sports sciences. The Galaxy project is developed through a collaborative effort across several universities and provides a web-based solution to perform genomic research using big data technologies (Goecks et al. 2010; Levine and Hullett 2002; Ohmann et al. 2015). The project aims to provide a standardized way for researchers to access complex processing algorithms which makes it possible for non-expert users to apply cutting edge analysis technologies to their data (Goecks et al. 2010). The system includes a sophisticated documentation solution which allows the storage and presentation of analysis results and documents at the same time the complete processing pipeline ensuring reproducibility of the research results (Goecks et al. 2010). The framework was build to be extensible and allows the inclusion of additional procedures through public repositories efforts (Blankenberg et al. 2014). This approach may be a model for sports sciences to address not only big data approaches for soccer tactics but more general analysis and data processing problems in other domains as well. Inevitable this will lead to increased collaborative efforts between sports and computer scientists as the sports science community at present lacks the required computational background.

## Conclusion

 In conclusion, exciting times are emerging for team sports performance analysis as more and more data is going to become available allowing more refined investigations. The adaption of big data technologies for soccer research may therefore provide solutions to some of the key issues outline above. Thus, by providing novel methods to analyze the data and a more comprehensive theoretical model and understanding of tactical team performance in elite soccer may be within reach. This implies however, that future soccer research will have to embrace a stronger multi-disciplinary approach. Performance analysts, exercise scientists, biomechanists as well as practitioners will have to work together to make sense of these complex data sets. As has been pointed out, most of the machine learning approaches presented were performed by computer science research groups. Accordingly, future collaborations between computer and sports scientists may hold the key to apply these complex approaches in a more relevant manner. In turn, relying increasingly on more complex data analysis techniques will also pose new challenges for future sports scientists. Therefore, university curricula will have be augmented to ensure that future students receive the required background training to be able to not only use these techniques but to have at least some understanding of their theoretical and computational underpinnings. The introduction of big data technologies will also require a discussions within the research community of how to share data and techniques across research teams. To make the new insights relevant for practice a tight interchange with practitioners is required. Finally, taking a broader view on the issue of big data and sports science the proposed model for tactical analyses in elite soccer might also prove beneficial for other sports science domains where data sizes are bound to increase as well and accordingly similar problems will surface.
